# Developing a community-centred malaria early warning system based on indigenous knowledge: Gwanda District, Zimbabwe

**DOI:** 10.4102/jamba.v8i1.289

**Published:** 2016-09-29

**Authors:** Margaret Macherera, Moses J. Chimbari

**Affiliations:** 1 Department of Environmental Science and Health, National University of Science and Technology, Zimbabwe; 2 College of Health Sciences, University of KwaZulu-Natal, South Africa

## Abstract

Malaria continues to be a major public health problem in Sub-Saharan Africa despite efforts that have been made to prevent and control the disease for many decades. The knowledge on prediction and occurrence of the disease that communities acquired over the years has not been seriously considered in control programmes. This article reports on studies that aimed to integrate indigenous knowledge systems (IKS) on malaria into the malaria control programme in Gwanda District, Zimbabwe. The studies were conducted over a 3-year period. Data were collected using participatory rural appraisals, key informant interviews, household interviews and workshops in three wards (11, 15 and 18) with the highest malaria incidence in Gwanda District. Disease livelihoods calendars produced by the community showed their knowledge on the relationship between malaria, temperature and rainfall, and thus an understanding of malaria as a hazard. Volunteer IKS experts willing to record the indigenous environmental indicators for the occurrence of malaria in the study area were identified by the communities. Indigenous environmental indicators for the occurrence of malaria were classified as insects, plant phenology, animals, weather and cosmological indicators. Plant phenology was emphasised more than the other indicators. A community-based malaria early warning system model was developed using the identified IKS indicators in two of the wards using the ward health team as an entry point to the health system. In the model, data on indicators were collected at the village level by IKS experts, analysed at ward level by IKS experts and health workers and relayed to the district health team.

## Introduction

Approximately 70% of recent disasters are weather related (Mercy Corps and Practical Action 2010). Natural hazards cannot be prevented and they are considered as disasters when vulnerable communities are caught unprepared (United Nations Development Programme [UNDP] & European Commission Humanitarian Office [ECHO] 2010; United Nations International Strategy for Disaster Reduction [UNISDR] 2005). Malaria epidemics are some of the most serious public health emergencies that confront health workers. They affect highly vulnerable populations with only limited immunity to malaria (WHO 2004). Epidemic malaria is a serious problem in semi-arid and highland areas in Africa. It is estimated that the epidemic of malaria causes between 12% and 25% of estimated annual worldwide deaths owing to malaria (Thomson *et al*. quoted in Komen *et al*. 2015). Early detection, containment and prevention of malaria constitute one of the four technical elements of the global malaria control strategy. The failure to immediately detect and control epidemics results in unacceptably high mortality and morbidity rates (Thomson *et al*. 2005). Malaria, being a vector-borne disease, is affected by variations in climatic factors and its occurrence can therefore be predicted using these climatic factors.

Malaria in Zimbabwe is highly seasonal and is caused by *Plasmodium falciparum.* The World Health Organization estimates that 50% of the country’s population is at risk of contracting malaria (WHO 2013; Kanyangarara *et al*. 2016). Fifty-four of the 59 rural districts in Zimbabwe have malaria levels which vary from very high and seasonal to sporadic (Ministry of Health and Child Care [MOHCC] 2008). The Ministry of Health and Child Care has a unit called the National Malaria Control Programme (NMCP) in its Department of Disease Prevention and control. This unit spearheads the prevention and control of malaria (MOHCC 2008). The NMCP uses the weekly surveillance system and a community-based early warning system that communicates public health events (Government of Zimbabwe [GOZ] & Ministry of Health and Child Welfare 2011). In general, there is inadequate use of meteorological data for malaria epidemic prediction at all levels (GOZ & Ministry of Health and Child Welfare 2011) probably because of inadequate coordination between the meteorological department and the Ministry of Health (GOZ & Ministry of Health and Child Welfare 2011). DaSilva *et al*. (2007) indicated that health services need better information on where and when outbreaks will occur. This information cannot be obtained from within the health sector and might need to come from meteorological or food security services (DaSilva *et al*. 2007). The health sector alone cannot develop an effective early warning system, especially with no readily available meteorological data. Hence, community capacity in weather forecasting using indigenous knowledge systems may provide complementary data in the development of such warning systems (DaSilva *et al*. 2007). The Zimbabwe Ministry of Health and Child Care has included the following two objectives in its programme for preventing and controlling malaria: (1) improvement of detection and timely control of malaria epidemics and (2) strengthening of community participation to maximise achievement of universal access to malaria control interventions (GOZ & Ministry of Health and Child Welfare 2011). The benefits of community participation in malaria control have been documented (Deressa, Olana & Chibsa 2005; WHO 2002). In our opinion, community participation in malaria control programmes maybe enhanced through the incorporation of the community’s local knowledge into the control programmes.

Several studies have shown the importance of climatic factors in the development of malaria early warning systems (Ceccato *et al*. 2007; Teklehaimanot *et al*. 2004; Thomson & Connor 2001; Thomson *et al*. 2005). Rainfall anomalies are widely regarded to be a major driver of inter-annual variability of malaria incidence in semi-arid areas of Africa (Thomson *et al*. 2005). A study conducted in Kenya that investigated the relationship between malaria and weather conditions concluded that rainfall shows the most predictive pattern for malaria transmission in the endemic study area (Sewe, Ahlm & Rocklov 2016), thus confirming the possibility to predict the occurrence of malaria using rainfall prediction.

The rainy season in Zimbabwe normally runs from mid-November to March (Muzari *et al*. 2014) and coincides with the malaria transmission season which peaks between February and May as a result of the preceding rains (Thomson *et al*. 2006). A model to analyse the spatial temporal role of climate in inter-annual variation of malaria incidence in Zimbabwe for the period 1988 to 1999 showed that high annual malaria incidence coincided with high rainfall and relatively warm conditions while low incidence years coincided only with low rainfall (Mabaso *et al*. 2006). The study demonstrated that mean values of temperature, rainfall and vapour pressure are strong predictors of increased malaria incidence. The communities’ ability to relate the occurrence of rainfall to malaria has also been indicated in Botswana (Chirebvu, Chimbari & Ngwenya 2013).

Given that local communities have the capacity to predict weather conditions using indigenous knowledge (Kalanda-Joshua *et al*. 2011; Mudzengi *et al*. 2013; Nethononda, Odhiambo & Paterson 2013; Shoko & Shoko 2013*),* there is potential for utilising such knowledge for the development of community-based malaria early warning systems. Community-based early warning systems (CBEWS) have been proved beyond doubt to save lives and reduce economic loses (Mercy Corps and Practical Action 2010). The same authors argue that CBEWS have shown that people are capable, resilient and able to protect themselves rather than becoming vulnerable. The German Committee for Disaster Reduction suggests that there exist four basic requirements to develop a community-based early warning system. These include individual and institutional knowledge of the threat, the ability to monitor and communicate change in the threat, disseminate information about the threat and the ability to respond to the threat (UNISDR 2006). In the context of malaria, as derived from the UNISDR requirements for a community-based malaria early warning system, this means that the people have to know the causes of the disease, its signs and symptoms and the season of occurrence. They should also be able to monitor and interpret indicators for the occurrence of the disease, disseminate warning information about the disease and be able to institute preventive measures and or solicit for assistance (UNIDSR 2006). Most of the documented community-based early warning systems focused on floods (Christian AID Malawi 2009; Gautam & Phaiju 2013; Lassa & Sagala 2013; Phaiju & Bhandari 2012; Van Loon 2010). The only documented community-based early warning system for communicable diseases that we know of is that of avian flu developed by CARE Philippines (2006). The possibility of developing a community-centred malaria early warning system has not been explored despite malaria being a weather-related disease. The current study was aimed at determining the prospects and challenges of utilising indigenous knowledge systems on malaria in the health and medical health care system in Gwanda District of Zimbabwe. The study led to the development of a framework for community-based malaria early warning system using indigenous knowledge systems. The possibility of its integration into the health and medical care system in the district was demonstrated.

## Methodology

### Study area

**FIGURE 1:**
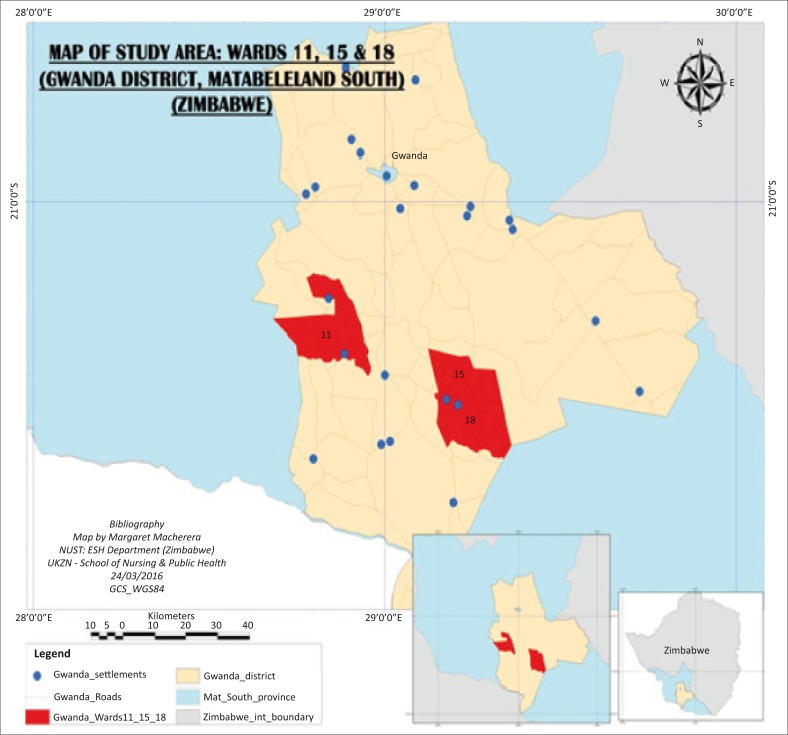
Map showing the study are wards 11, 15 and 18 in Gwanda District Zimbabwe.

This study was conducted in Matabeleland South Province south of Gwanda District, Zimbabwe. Gwanda District is 123 km south of Bulawayo and has borders with Umzingwane District in the West, Insiza District in the East and Beitbridge and Botswana in the South (Figure 1). The district has both rural and urban wards.

A ward is an administrative area within a district comprising several villages. The number of villages in a ward is not fixed and it varies from ward to ward. On average, a village consists of 100 households. At the ward level, there is a ward development committee (WDC), comprising the elected ward councillor, the kraal heads (traditional leaders subordinate to chiefs) and representatives of village development committees (VDC). Each village has an elected VDC and a headman (traditional leader subordinate to the kraal head).

Health issues at the ward level are administered through a ward health team which reports to a district health team (DHT) managed by a district health executive (DHE). The DHT in turn reports to a provincial health team (PHT) that is managed by a provincial health executive (PHE). The PHE reports to national level (Government of Zimbabwe [GOZ] 2012).

There is unstable malaria in nine wards in the southern part of Gwanda District. The wards with the highest incidence of malaria are 11, 15 and 18 (District Health Information, 2013).

### Methods

Three wards with the highest malaria incidence (Ward 11, 15 and 18) (GOZ & Ministry of Health and Child Welfare 2013) were purposively selected for the study. Pre-tested key informant interview guides and focus group discussion (FGD) guides were used for data collection. Participatory rural appraisals (PRAs) were also conducted and the data were validated during community workshops. This study was divided into three components. Data collection was conducted from 2013 to 2016. The data collection methods were the same for all the components of the study. The methods for each component are described below. The study focused on the determination of the existing indigenous knowledge systems on malaria in the study area including prediction, prevention and treatment of malaria.

Concurrent multilevel data analysis was conducted and the data from the various sources was synthesised. The credibility, transferability and trustworthiness of the data were covered by triangulation of sources and convergence of the themes using literature (Polit & Beck 2012).

#### Indigenous knowledge systems for management and prevention of malaria

Participants for FGDs were identified through the ward councillors who provided a list of the elderly men and women and their age groups. The elderly people were ranked according to their age groups starting with the eldest until 12 participants were obtained ensuring equal representation by gender. The discussions were conducted at ward centres during times convenient to the participants. Key informant interviews were conducted with traditional leaders, traditional healers and the elderly who were purposively sampled. The number of key informants interviewed in each ward depended on the number of leaders and healers in each ward. Where the leaders or healers were not available, the elderly aged 50 years and above who were renowned custodians of indigenous knowledge systems and were not included in the FGDs were interviewed. A total of 28 key informants across the three wards were interviewed. These data were complemented by PRAs conducted in the study area. Communities were asked to construct disease calendars in order to find out their understanding of the disease, its causes and the season of occurrence. This was important to ensure that the study participants and the researchers had common understanding of malaria as a hazard. Trend analysis for the occurrence of malaria was also documented during FGDs to further confirm the community’s understanding of the disease.

#### Indigenous environmental indicators for predicting occurrence of malaria

Key informant interviews, PRAs and FGDs were used to collect data on indigenous environmental indicators (IEI) used by communities to predict occurrence of malaria. Three follow-up workshops were conducted with 15 indigenous knowledge specialists from each of the three wards to verify the findings from previous data collection sessions. At this stage, the data obtained from the community were presented to them for verification. Corrections were made where anomalies were noted. This part of the study resulted in the documentation of the indicators used by the community to predict the occurrence of malaria in the study area.

#### Development of a community-based malaria early warning system

After the documentation of indicators for the occurrence of malaria was completed, volunteers willing to carry out observations documenting the indicators were identified. The documentation of the indicators was important in order to enable analysis of the observations at ward health team meetings. This was to enable the integration of these observations into the existing malaria control programme.

Workshop participants were asked to identify volunteers to observe the indicators identified in component (2) of the study. The volunteers were not necessarily involved in the malaria control programme prior to the study. Two volunteers were selected per village. It was agreed that observations would start in the month of July soon after the workshops and analysis would be done in October. The observers were trained on how to record and they were given books in which to record the indicators. The books were kept by the ward secretaries in Wards 15 and 18 and by the ward coordinator in Ward 11. Two ward health team meetings were conducted in each ward. The purpose of the first meeting was to introduce the idea to the Ward Health Team (WHT) and also for the volunteers to meet the WHT. The second meeting was to analyse the data on indicators collected by the volunteers. Two meetings were also held with the DHE to introduce the system and to discuss the analysis from the ward level.

## Results

### Indigenous knowledge systems on malaria

**FIGURE 2:**
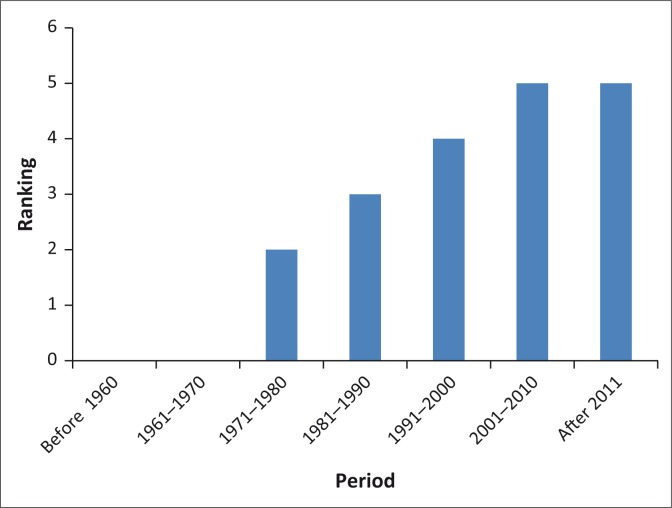
Perceived malaria trends 1960–2014: ward 15.

In all the wards studied, the main vernacular names used for malaria were ‘uqhuqho’ or ‘inyongo’ and the name ‘umkhuhlane wemiyane’ was mentioned in one of the FGDs in Ward 11. The name uqhuqho means shivering while umkhuhlane wemiyane means disease caused by the mosquito. Indigenous knowledge systems on the prediction, treatment and prevention of malaria were assessed. The assessment showed the existence of this knowledge in the area. The communities were able to relate the occurrence of mosquitoes and rainfall to malaria. They made a trend analysis of malaria from 1970 to 2011 and that of rainfall and temperature from 1960 to 2011. FGD trends analyses for malaria were done in Ward 15 for the period 1960–2014. The year 1960 was selected because the oldest participant in the group had stayed in the area since 1960. The participants in the FGDs were asked to rank the occurrence of malaria on a scale of 1–10 for each 10-year period from 1960 to date. Figure 2 shows how the occurrence of malaria was ranked by the villagers. The *x*-axis shows the ranking suggested by the FGD participants for the occurrence of malaria, rainfall and temperature levels on a scale of 1–10, with 10 being the highest.

**FIGURE 3:**
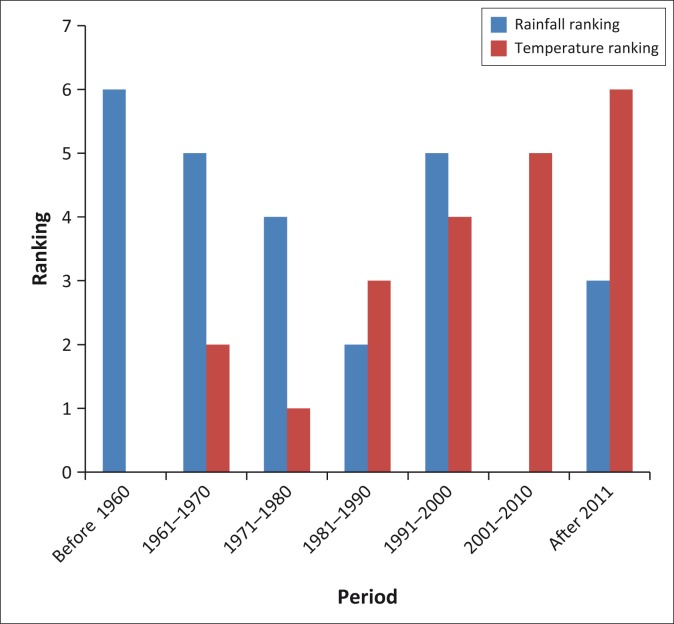
Perceived temperature and rainfall trends 1960–2014: ward 15.

Rainfall and temperature trends were also ranked using the same method as that used for ranking malaria. Figure 3 shows how the FGD participants ranked temperature and rainfall during the same period from 1960 to date. According to the villagers, malaria was not a problem in the 1960s. They started noticing the problem in 1971 and thereafter it has been increasing.

The study participants acknowledged that before the 1960s, the area received much rain and from 1971 the rain became erratic and the temperatures began to rise.

**FIGURE 4:**
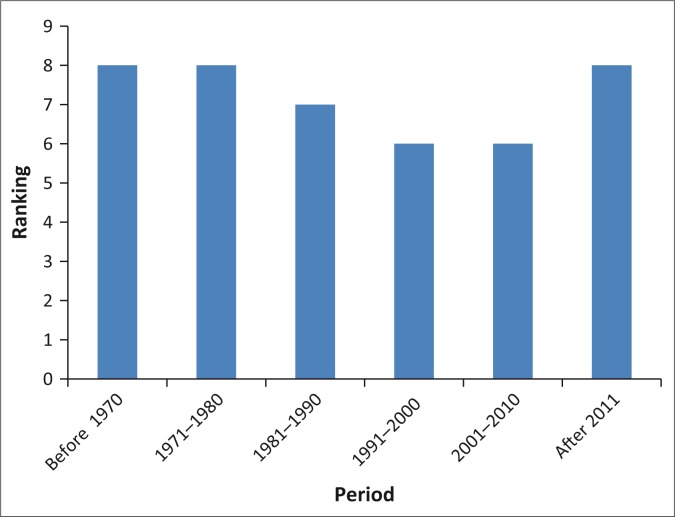
Perceived malaria trends 1970–2014: ward 11.

Figure 4 shows malaria trends as perceived by the Ward 11 community for the period 1970–2014. Participants who had stayed in the area the longest had been there since 1970. The occurrence of malaria was shown to have been above 50% on a scale of 1–10 with the highest being between 1970 and 1980 and also from 2011–2014.

The construction of a community disease calendar showed that the community understood the malaria hazard. This activity was done during the PRAs.

**FIGURE 5:**
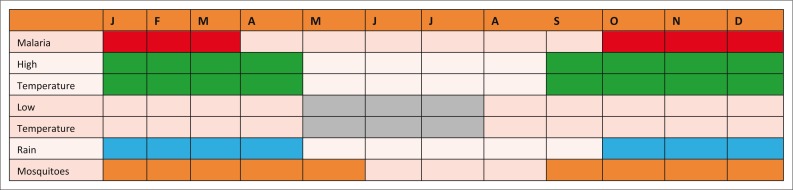
Community Disease Calendar from the Participatory rural appraisals.

Figure 5 shows the calendar drawn by the communities during PRAs.

The occurrence of malaria coincided with the time when people observed high numbers of mosquitoes in the area. It is during this time that the people also said they experienced high rainfall and high temperatures. The calendar showed that when there were low temperatures, mosquitoes were absent, rainfall was absent and malaria was also absent; they used this information to predict the occurrence of malaria.

### Indigenous environmental indicators for the occurrence of malaria

Participants of FGDs had consensus on the indicators that they used to predict the occurrence of malaria in the study area which concurred with those mentioned by the key informants. Plant phenology was indicated as the most used indicator in predicting malaria. Other indicators mentioned include behaviours and movement of birds, insects and animals. The findings revealed that most of the indicators that the communities used for the prediction of malaria were those that occurred from September to November. Communities expressed that these indicators worked for them. They however said that sometimes they would miss the indicators because observation was on an individual basis. The knowledge was communicated to the younger generations through community meetings. Meteorological indicators including wind patterns, direction and variation were mentioned as monitoring indicators for malaria. The key informants expressed that the indicators were reliable. This concurred with the findings from the FGDs. The FGD participants mentioned that the community had informal regulations that ensured the conservation and preservation of the environmental indicators that they used to predict malaria.

They singled out trees like *Boscia albitrunca* (Burch.) Gilg & Gilg-Ben and *Colophospermum mopane* (J.Kirk ex Benth.) J.Kirk ex J.Léonard as some of the big trees that should be conserved. One of the ways in which the communities protected important trees was the general rule that all trees that are to the east of a homestead should not be cut as they serve as windbreaks. The rule however applies to all trees and not necessarily those that are used as indicators of the occurrence of malaria.

Participants of FGDs indicated that there were very few animals left in their wards. They, however, indicated that lions and elephants were still present in their areas and were used as indicators for malaria. They expressed that if lions or elephants pass through the villages at night during the month of September, it means that the coming malaria season is going to be bad. The participants also said that people were not allowed to kill animals of their totem. This helped them to protect perceived important animals.

### Developing the early warning system

The indigenous environmental indicators for the occurrence of malaria were corrected and endorsed by workshop participants in all the three study wards. They agreed that it was possible to develop a community-based malaria early warning system using the IKS indicators. The participants were asked to select observers of the indicators. In all the wards, the workshop participants volunteered to observe the indicators and to analyse them. The demographic characteristics of the observers are shown in Table 1.

**TABLE 1:** Demographic characteristics of observers.

Ward Number	Males	Females
11	8	7
15	11	4
18	10	9
**Total**	**29**	**20**

The ages of the observers ranged from 23 to 60. The majority of the observers (80%) were aged 50 years and above. The younger members were selected to facilitate documentation and also to ensure sustainability. The knowledge was mainly with the elderly. Forty-one per cent of the observers were females.

### Documentation of indicators

**FIGURE 6:**
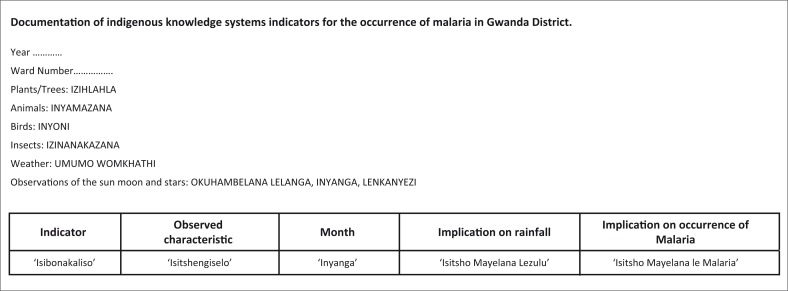
Format for documentation of indigenous knowledge systems indicators for the occurrence of malaria.

There was no formal documentation of indicators that existed in all the three wards prior to the study. Therefore, participants agreed on a format for the documentation of indicators as shown in Figure 6.

### Generic structure of the community-based malaria early warning system

Participants agreed that after documenting the indicators, they would analyse the data at the ward level to determine the implications of the indicators on occurrence of malaria. They agreed to involve the Ministry of Health so that their ideas could be incorporated into malaria control activities. Figure 6 shows the generic structure of the system suggested by the community. Wards 15 and 11 suggested that the ward-level malaria control committee would be the ward health team while the participants in Ward 18 agreed that their ward-level malaria control committee would be the ward disaster risk management committee. In all the wards, workshop participants agreed that they were going to be the IKS specialists. The volunteer teams comprised community health workers. The community health workers would carry out surveillance and health education while community research assistants would identify breeding places and assist in case finding and health education during malaria outbreaks.

The participants from Ward 15 agreed that the IKS experts would submit their data to the ward secretary. The record book for Ward 18 was kept by the councillor while that for Ward 11 was kept by the ward coordinator who was also assigned as the contact person for the early warning system project. Village meetings were held to allow input of indicators from other villagers in the ward who were not part of the IKS team. Data on observed indicators were submitted to the teams during village meetings. They also suggested integrating the observations they made for malaria with observations for agriculture.

### Operationalisation of the system

**FIGURE 7:**
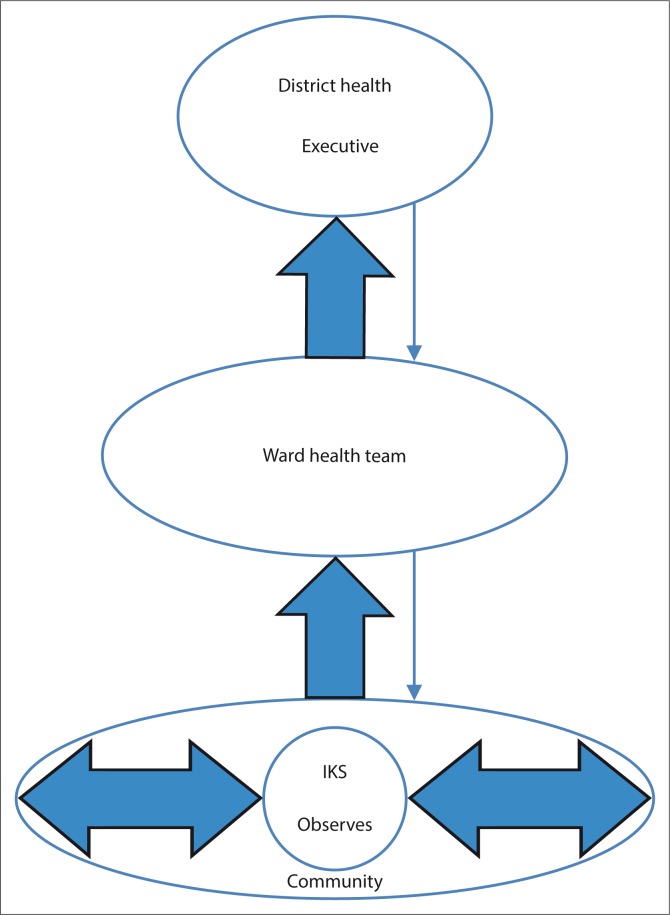
Generic structure of the community-based malaria early warning system.

The ward health teams in the study wards accepted the structure of the community-based malaria early warning system (CBMEWS) as shown in Figure 7 as they said it utilised existing structures. As such, the existing committees were given the additional responsibility of observing IKS environmental indicators formally. They also appreciated the fact that the elderly people felt relevant and useful in the community as observers of these indicators. The initiation of observations for both Wards 15 and 11 was agreed to be during the month of August. The DHE however expressed that they would want the IKS experts to start the observations in July and inform the DHE in September. Analysis of the observed indicators was to be during the third quarter ward health team meeting which is usually held during the last week of September or the first week of October. Information obtained from the analysis was disseminated during the month of October to the general community and to the DHE. The DHE acknowledged that the district did not have a well-defined method for the prediction of malaria. Meteorological data are not used in the district because of lack of capacity to collect and analyse such data. They also indicated that the district no longer uses threshold values because they are in the pre-elimination phase. One positive case is considered to be an outbreak. They therefore accepted the utilisation of IKS for malaria prediction. The DHE however also identified barriers to the utilisation of IKS which included climate change. They acknowledged that the weather is erratic and the disease pattern is also changing, making it difficult to predict. They said this could be overcome if communities did not rely on one indicator but rather on a combination of indicators for determining the weather forecast. They noted that the formalisation and inclusion of the community-based malaria early warning system into the health system may require input from policy makers who are at a higher level than the district level. However, a pilot of the system in the district was feasible.

### Action to be taken after predictions

The ward health team members agreed on the action that the community should take in the event of the occurrence of malaria being predicted. This included health education through the village health workers, identification of potential breeding places and covering the places. They also said they would conduct awareness campaigns to inform people on the signs and symptoms of the disease, the need to seek early treatment and the need to allow spraying of their houses by the Ministry of Health and Child Care. The DHE would incorporate the analysis by ward health teams into their plans for malaria control and these would be discussed in the level 2 malaria control training which is conducted during the first week of October every year.

The warning is called off in December because all the signs will have been observed and the malaria season will have started. The evaluation of the system is conducted during the first quarter ward health team meeting after the malaria season. The evaluation activities included the accuracy of the predictions, the effectiveness of the communication system and the timeliness of the response activities.

## Discussion

The various components of this study resulted in the development of community-based malaria early warning systems in the three wards that were studied. The methodology was mainly derived from the four elements of early warning systems, namely risk knowledge, monitoring, warning communication and response capability (UNISDR 2009).

The study participants from the three wards in Gwanda District understood that malaria is transmitted by mosquitoes. This was displayed by one of the commonly used names of the disease umkhuhlane wemiyane literally meaning a disease of the mosquito. This could be attributed to the health education conducted by the Ministry of Health and Child Care in the district every malaria season. The participants also displayed a good knowledge of malaria in terms of the signs and symptoms. The other commonly used name ‘uquqo’ explains how the disease presents. The third name inyongo that was mentioned by fewer respondents however indicated that there were still some misconceptions that existed about the disease. The name describes a condition experienced by some people who vomit bile after eating sweet things. Similar findings on this misconception were also reported by Ngarivhume *et al*. (2015) in the Chipinge district of Zimbabwe. According to the UNISDR (2005), it is essential that communities understand the risks so that they will be able to respect the warning and know how to react. In this study, it was essential to ensure that the term malaria was interpreted to mean the disease transmitted by mosquitoes. It was also essential at this stage to clear all misconceptions that were identified.

A deeper understanding of the risk was demonstrated by the community knowledge of the season of occurrence of malaria. This was shown by the community disease calendar produced during the PRAs and the trend analyses done during the FGDs. According to the World Health Organization, the main determinants of the intensity of malaria transmission are the distribution of the main vector species, altitude, temperature, humidity, rainfall and the distribution of rural urban populations (WHO 2006). Previous studies have shown the importance of climatic factors in the development of malaria early warning systems (Ceccato *et al*. 2007; Teklehaimanot *et al*. 2004; Thomson & Connor 2001; Thomson *et al*. 2005). The communities demonstrated knowledge of the relationship between malaria temperature, rainfall and the occurrence of mosquitoes. This knowledge that the ordinary community members have is not considered in malaria control programmes.

According to the World Conference on Disaster Reduction, January 2005, Kobe, Japan, effective early warning systems must be embedded in an understandable manner and be relevant to the communities which they serve. In this study, indigenous environmental indicators were used to predict the occurrence of malaria. These indicators have always been used by these communities to predict the disease but have not been formally recorded or discussed. The trend analyses that the communities made for rainfall, temperature and malaria showed the dynamic nature of malaria as a hazard and the vulnerabilities that arose from climate variability.

The selection of indigenous knowledge specialists who collected data on indicators of rainfall facilitated continuous monitoring and forecasting of the occurrence of malaria. The times for the analysis of the collected data coincided with the Ministry of Health’s planning period for malaria. This made integration feasible since the findings from the community could be presented to the DHE at the right time. The age range of the observers selected by the communities allowed for sustainability. Since the custodians of this knowledge were the elderly, inclusion of younger people in the team would mean that the knowledge will be passed down to the younger generation. The groups of observers also included women who are more vulnerable to malaria especially during pregnancy and are also care givers at the household level. The UNIDSR (2006) definition for a people-centred early warning system puts the affected people at the centre and acknowledges that they can contribute towards reduction of their vulnerability to hazards. The Sendai workshop in March 2015 emphasised the engagement with relevant stakeholders, including women, children and youth, persons with disabilities, indigenous groups and immigrants in the development of early warning systems (UNISDR 2015). This was catered for in the current study.

The communities that were studied in Gwanda acknowledged that the indicators that they observed could also help them in deciding the types of crops to grow in the coming season. This means that they would be able to predict drought or floods as well as malaria at the same time. This was made possible by inclusion of officials from the Ministry of Agriculture in the ward health teams (GOZ 2012). The United Nations International, Strategy for Disaster Reduction acknowledges that disaster risk reduction practices need to be multi-hazard and multi-sectoral (UNISDR 2015).

The communication channels for the warning were determined by the communities. The communication of the indicators was two directional – from the observers to the general population and also from the general population to the observers through the village meetings. After analysis of the indicators, the warning is communicated to the people through the village health workers, the observers and the health workers. Members of the ward health team also participate in the warning communication. The UNISDR platform for the promotion of early warning requires that multiple communication channels be utilised to ensure that as many people as possible are warned (UNISDR 2006). The communication channels used in this study already existed and were well established within the community structures. The ward health team is an existing structure which takes care of all the health needs at the ward level. It is mandated by the Zimbabwe Manual for Local Government (GOZ 2012). The meetings that are used to analyse the data and disseminate the warning are those that are already scheduled like the quarterly ward health team meetings and the village meetings. The WDC meetings are also utilised since the councillor who chairs these meetings also chairs the ward health team (GOZ & Ministry of Local Government 2000).

In this study, the participants determined activities that would be necessary in the event of a warning being issued. The activities included the destruction of potential breeding places for mosquitoes, and health education among others. This is in line with the UNISDR (2015) recommendation that the community should know how to react when a warning is issued. The education programmes in response to the warning at the ward level are incorporated into the existing health education programmes under the Ministry of Health and Child Care. The ward health team appreciated that there were things that they were not able to do at their level. Those are taken care of by the next level when the predictions are communicated to the DHE through the district health team meetings. In this system, there is a local bottom-up approach as well as a top-down approach in the form of feedback and reinforcement of the warning system from the DHE (UNISDR 2015).

## Conclusion

Our study demonstrated the possibility of developing a community-based malaria early warning system using indigenous knowledge systems. We showed how the system can be integrated into the conventional health system.

Based on our work, we recommend the following steps in developing a community-based malaria early warning system. These steps may be adapted to suit administrative structures in the area of implementation but for Zimbabwe only small modifications may be necessary:

- Step 1: Determination of the community knowledge on malaria. This should include the knowledge on the causes and the signs and symptoms and the season of occurrence.
- Step 2: Identification of indigenous environmental indicators for the occurrence of rain as a precursor to the occurrence of malaria.
- Step 3: Identification of observers and consensus on what to observe and the recording format for the observed indicators.
- Step 4: Consultation with the ward health team and the DHE to ensure that the system is integrated into the conventional health system.
- Step 5: Collection of data on indicators by the observers.
- Step 6: Analysis of data at the ward level with the ward health team.
- Step 7: Communication of results to the DHE and the community and the initiation of action by the community and the ward health team and the DHE.
- Step 8: Evaluation of the system after the malaria season by the ward health team and the observers.
